# Searching the Grey Literature: A Handbook for Searching Reports, Working Papers, and Other Unpublished Research

**DOI:** 10.5195/jmla.2019.640

**Published:** 2019-04-01

**Authors:** Gerald Natal

**Affiliations:** Mulford Health Science Library, University of Toledo, Toledo, OH, gerald.natal@utoledo.edu

The author of *Searching the Grey Literature: A Handbook for Searching Reports, Working Papers, and Other Unpublished Research*, librarian/researcher Sarah Bonato, refers to the topic of her book as “the behemoth of all that *other* literature,” which hints at the reason why a book on the topic is warranted and sets the tone of voice for an enjoyable read. Many librarians might be familiar with the blanket term, but grey literature covers a wide variety of sources, and knowing which types to search and under what circumstances might not be so familiar. This book can help in making those decisions. This book is written in a straightforward, practical style, and the material is enlivened by the use of quips and quotes. Readers should come away with a basic knowledge of the range and complexity of grey literature, as well as where it falls within the larger picture of information.

Given that the term is broadly defined, this book provides several definitions for grey literature, along with an overview of recent research on the subject. The book is arranged in such a way that it can be used as teaching material: each chapter provides learning outcomes and aids for decision making. Sources for searching for grey literature are provided, along with the scope of each resource, strengths and weaknesses, tips for searching, and several examples of grey literature searches. All this information can help to facilitate discussions with information users about the selection and evaluation of grey literature resources.

The book does a good job of explaining the reasons for an increase in the importance of grey literature. It details how grey literature offers value, describes how it compares with other types of information, and, in general, makes a great case for including grey literature in research (with particular importance given to inclusion in systematic reviews). It also contains methods for determining the quality of grey literature and gives the benefits and drawbacks of various grey literature resources. Examination of several of the resources under evaluation proved observations and suggestions that the author made to be accurate. The section on developing a grey literature search plan with different scenarios is particularly useful. Anyone unfamiliar with nuanced searching in Google and Google Scholar or doing a systematic review should find those sections useful as well.

A previous book on the subject from 2010, *Grey Literature in Library and Information Studies* by Dominic Farace and Joachim Schöpfel, compiled a decades’ worth of research on grey literature for librarians and information professionals. *Searching the Grey Literature* attempts to capture the present state of grey literature and current relevant resources and to make sense of what the author refers to as the “elephantine wellspring” of grey information. Until a uniform set of standards for locating grey literature is created, the information contained in this book should provide a welcome stopgap by suggesting best practices, methods, and approaches. The subject matter of the book should have wide appeal but may be of particular interest to anyone in the health and social sciences fields, and especially anyone involved in creating systematic reviews.

